# Highly efficient coherent conformal projection system based on adaptive fiber optics collimator array

**DOI:** 10.1038/s41598-019-39304-0

**Published:** 2019-02-26

**Authors:** Dong Zhi, Yanxing Ma, Rumao Tao, Pu Zhou, Xiaolin Wang, Zilun Chen, Lei Si

**Affiliations:** College of Optoelectronic Science and Engineering, National University of Defense Technology, Changsha, 410073 China

## Abstract

Coherent beam combination (CBC) of fiber laser array, which is a promising approach to overcome the power scaling limitations of the single fiber laser and to achieve high-brightness laser with good beam quality, can be employed in coherent conformal projection system (CCPS) for beam projection and free space laser communication system with enormous potential. To date, all the demonstrated CBC system through several kilometers atmospheric turbulence have employed collimated beams, which significantly decrease the final CBC effect because of the inefficient wavefront overlap. On the other hand, focused CCPS has lots of advantages over collimated projection system since the wave front of the projected beam is closer to that of a convergent spherical wave. In this paper, we design and manufacture focused CCPS based on an adaptive fiber optics collimator array for the first time, which is achieved by introducing controllable spherical aberration through the adaptive fiber optics collimator. A CBC system is setup to evaluate and validate its performance. Results show that compared with the collimated CCPS, the energy portion of the central lobe could be increased by a factor of 44%, which agrees well with the theoretical analysis.

## Introduction

As a promising approach to overcome the power scaling limitations of the single fiber laser, coherent beam combination (CBC) technique can achieve high-brightness laser with good beam quality^1–3^, while realizing various useful applications including phase distortion correction^4^, beam steering^5,6^ and wavefront shaping^7–9^, which can be used to surmount the challenges like the phase distortion (mainly including piston and tilt phase aberrations) of the combined fiber laser array after propagating through a turbulent atmosphere^10^. Generally, piston phase errors can be corrected by applying active phase-locking algorithms (dithering algorithm, SPGD algorithm *et al*.) on phase modulators in the CBC architecture^2–7,11^, and tilt deviation compensations can be realized by beam pointing or beam steering techniques, which utilize various complex devices including MEMS mirrors, liquid crystal phased array, Pancharatnam phase devices, and so on^12–14^. Adaptive fiber optics collimator (AFOC) has been proved to be an effective and simple approach to realize the tip-tilt phase compensation for fiber laser array combining system^5,15,16^. The coherently combined fiber laser power delivering system that employs AFOCs, which could be called as coherent conformal projection system (CCPS), enables shaping the phase of array beams into a discrete spherical one by tip-tilt phase controlling and then project the array beams to the same target^15,16^, has great advantages in the near-field propagation range (usually less than several kilometers)^17^. Moreover, CCPS also provides efficient performance in mitigation of atmospheric turbulence effects compared with traditional beam director with a monolithic mirror^18^.

The collimated CCPS, which could only compensates the piston and tilt wavefront aberrations, is ineffective to overcome the higher order phase distortions, such as defocus phase aberrations^7,19^. The focused CCPS, in which each AFOC transmits a focused beam with the same focusing distance, can manipulate the spherical phase of each beam and enables the array beams focus on the target simultaneously, which has advantaged superiority in improving power delivering efficiency^17^.

In recent years, collimated CCPS through the atmospheric propagation has been theoretically analyzed and experimentally demonstrated^20–22^. However, few attentions have been concentrated on the focused CCPS. Although the averaged intensity distribution of focused CCPS through turbulent atmosphere has been studied analytically^17^, focused CCPS has not been studied experimentally ever before. In this paper, we design and manufacture focused CCPS based on an adaptive fiber optics collimator array for the first time, and we present a series of CBC experiments using this home-made focused CCPS, which demonstrated effective improvement when focused CCPS was employed. Besides, the experimental results are discussed with theoretical analysis.

## Results

**Figure 1:**
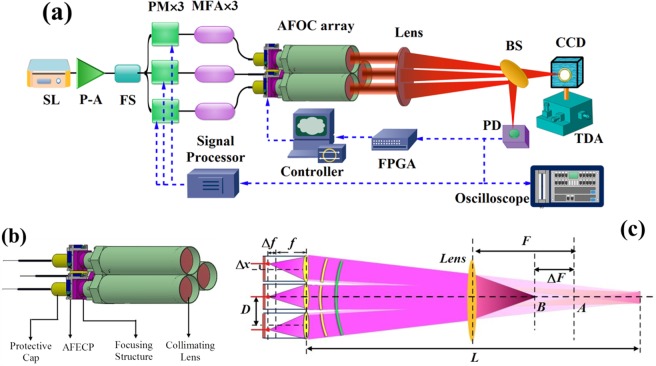
(**a**) The working schematic diagram of CBC experiment platform of home-made focused CCPS, (**b**) The structural diagrams of home-made focused CCPS, (**c**) The experimental schematic diagram of focused CCPS.

The experimental setup of the home-made focused CCPS is illustrated in Fig. 1(a). A single frequency Yb-doped fiber laser centered at 1064.15 nm is employed as the seed laser (SL), which delivers an output power of 80 mW and then is boosted to almost 400 mW by a pre-amplifier (P-A). The amplified laser is split into 8 channels by a fiber splitter (FS) and three channels are chosen in the experiment. Phase modulators (PM) are inserted into each channel to provide active and accurate piston phase control. A mode field adaptor (MFA) is used to connect the output fiber (core diameter of 10 μm) of the PM with the large mode area fiber (core diameter of 20 μm). Large mode area fiber is employed in our system for the greater output power handling capacity (usually upon kilowatt level^23^) than single mode fiber applied in previous demonstrations, for example, in refs^5,15^. Then each output fiber of the MFA with core/cladding diameter of 20/400 μm is spliced with a fiber end-cap, which is inserted in an home-made AFOC. Finally, the three laser beams output from AFOCs are focused together to the far field through a focusing lens with focal length (*F*) being 2 m. A beam splitter (BS) with splitting ratio of 50:50 is located in the beam array propagation path to split the combined laser beam into two parts. As depicted in Fig. 1(a), the transmitted part is collected by a CCD camera, which is fastened on a three-dimensional adjustor (TDA), to observe the combined beam patterns at different focusing points, while the reflected part is detected by a photonic detector (PD) to observe the collected light intensity signal and provide feedback data to the phase control systems, which contain piston phase control system and end-cap/tilt control system. The basic principle of the phase control systems is to extract the phase error information among the array beams from the voltage signal received by the PD with bandwidth of 150 MHz, and to greatly reduce the phase differences among the array beams in real time through reasonable evaluation function and optimization control algorithms. In the following CBC experiments, the piston and end-cap/tilt phase control systems are accomplished through a signal processor and a field programmable gate array (FPGA) processor, respectively. For the optimization control algorithms, single frequency dithering algorithm^11^ with 1 MHz iteration frequency and SPGD algorithm^6^ with 400 Hz iteration frequency are individually utilized to realize the piston phase control and the end-cap/tilt control. Moreover, the additional indication is that the diameter of each collimated beam and the distance of adjacent beamlets (*D*) are about 14.5 mm and 50 mm in our experimental setup.

**Figure 2:**
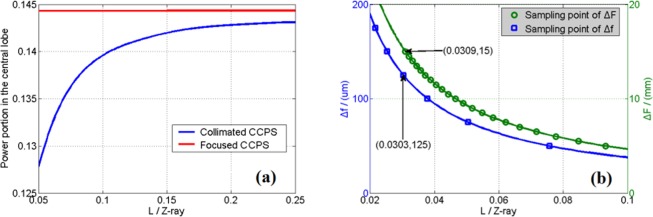
(**a**) The power portion in the central lobe as a function of propagation distance by numerically calculations, (**b**) The sampling points of Δ*F* and Δ*f* that can be chosen for experiment.

The structural diagram of focused CCPS is shown in Fig. 1(b). The focused CCPS, which is the subject of a pending patent application, consists of three AFOCs. Each AFOC is made up of a protective cap, an adaptive fiber end-cap positioner (AFECP), a focusing structure and a collimating lens. By utilizing the mechanism of gear and ratch with accuracy of 25 μm, the focusing structure can finely tune the position of fiber laser exit end along the optical axis. When the distance between the collimating lens and the fiber laser exit end becomes longer than the focal length, the emitting beam will no longer be a collimating beam and will focus to a definite distance. The AFECP, which can support high output power with preserved near-diffraction-limited beam quality, adopts two perpendicularly located piezoelectric stacks actuators to accurately position the fiber end-cap and control the tilt angle of the emitting beam, just as the presentations in ref.^16^. To explain the experimental principle clearly, an optical and mechanical schematic diagram of focused CCPS is depicted in Fig. 1(c). Normally, the fiber laser exit end locates at the focal point of the collimating lens with focal length of *f*. At this moment, the emitting beam from AFECP is collimated and the projection system corresponds to the collimated CCPS. When the location of the fiber laser exit end deviates from the focal point with a tiny distance of Δ*f* by regulating the focusing structure, the emitting beam becomes a focused beam and focuses to a definite distance of *L* = *f *^2^/Δ*f*. Under this circumstance, in order to make the array beams a high-degree overlapping, the laser exit end should be adjusted with an off-axis displacement of Δ*x* = *f*⋅*D*/*L*. The value of *L* is too large (usually over hundred meters) to be realized in laboratory. To reduce the experimental distance, we exploit a focusing lens with focal length of *F* to simulate a long distance propagation effect. As shown in Fig. 2(b), if the array beams transmit collimated to the focusing lens, the beams will be focused to the focal point *A*. when the array beams are defocusing with Δ*f*, the position of the overlapped array beams will shift from point A forward to point *B* with a distance of Δ*F*, which is calculated to be *F*^2^/(*F* + *L*). Thus by accurately controlling the values of Δ*f* and Δ*F*, we can experimentally simulate and validate the focused CCPS within a short distance in laboratory.

In the experiment, the far-field of the beam array is measured by the CCD camera, which is fastened to a three-dimensional adjuster as depicted in Fig. 1(a). The position of the CCD along the optical axis can be accurately controlled by adjusting this three-dimensional adjuster, which has accuracy of 0.5 mm and range of 15 mm. The focal point position after the lens represents the infinite propagation distance of the beam array, which is called far-field. When the array beams propagate in the near-field range, the corresponding target position should move forward from the focal point of the lens. By regulating the three-dimensional adjuster, we can control the CCD to the target position, which is equivalent to the near-field propagation distance.

Before the experiments carried out, by theoretical analysis and calculations, the power portion in the central lobe, which is known as beam propagation factor (BPF), as the function of normalized propagation distance is shown in Fig. 2(a). Here the parameter Z_ray = (π*D*_a_^2^)/(4*λ*), which is calculated to be 8.56 km, generally can be used to represents the far-field effectively^17,20^. Here *D*_a_ is the aperture diameter that contains all beams and measured to be 107.7 mm in our experiment. From Fig. 2(a), we conclude the ideal power portion in the central lobe of collimated CPS decreases faster as the focusing distance less than 0.1 times of Z_ray. To validate the variation trend of the power portion in the central lobe along with the propagation distance, we should choose at least three sampling points with the same interval to do the experiments based on collimated CCPS. Furthermore, to verify the superiority of focused CCPS to collimated CCPS, we should choose a specified short propagation distance *L*. When choosing this *L*, the mechanical accuracy of the focusing structure and the movement range of the CCD should be taken into consideration. By considering the limitations aforementioned, the sampling points of Δ*F* and Δ*f* that can be chosen for experiment are depicted in Fig. 2(b). To do the experiment at the specified short propagation distance, Δ*F* should be chosen the largest 15 mm by the measurement range limitation of the adjuster with the equivalent propagation distance of 0.0309 times of Z_ray. At the same time, we should select a corresponding sampling point of Δ*f*, which represents the same propagation distance, to do the comparative experiment based on the focused CCPS. Considering that the mechanical accuracy of the focusing structure is 25 µm, we can set Δ*f* to be 125 µm with the equivalent distance of 0.0303 times of *Z*_ray, which is a quite approximation. After all things considered, three sample points are chosen with their specific parameters listed in Table 1 below.

**Table 1:** Parameters of sampling points.

Sampling Points	1	2	3
Δ*F* (mm)	5	7.5	15
*L*/*Z*_ray	0.093	0.062	0.031
Δ*f* (µm)	40	61	122.4

**Figure 3:**
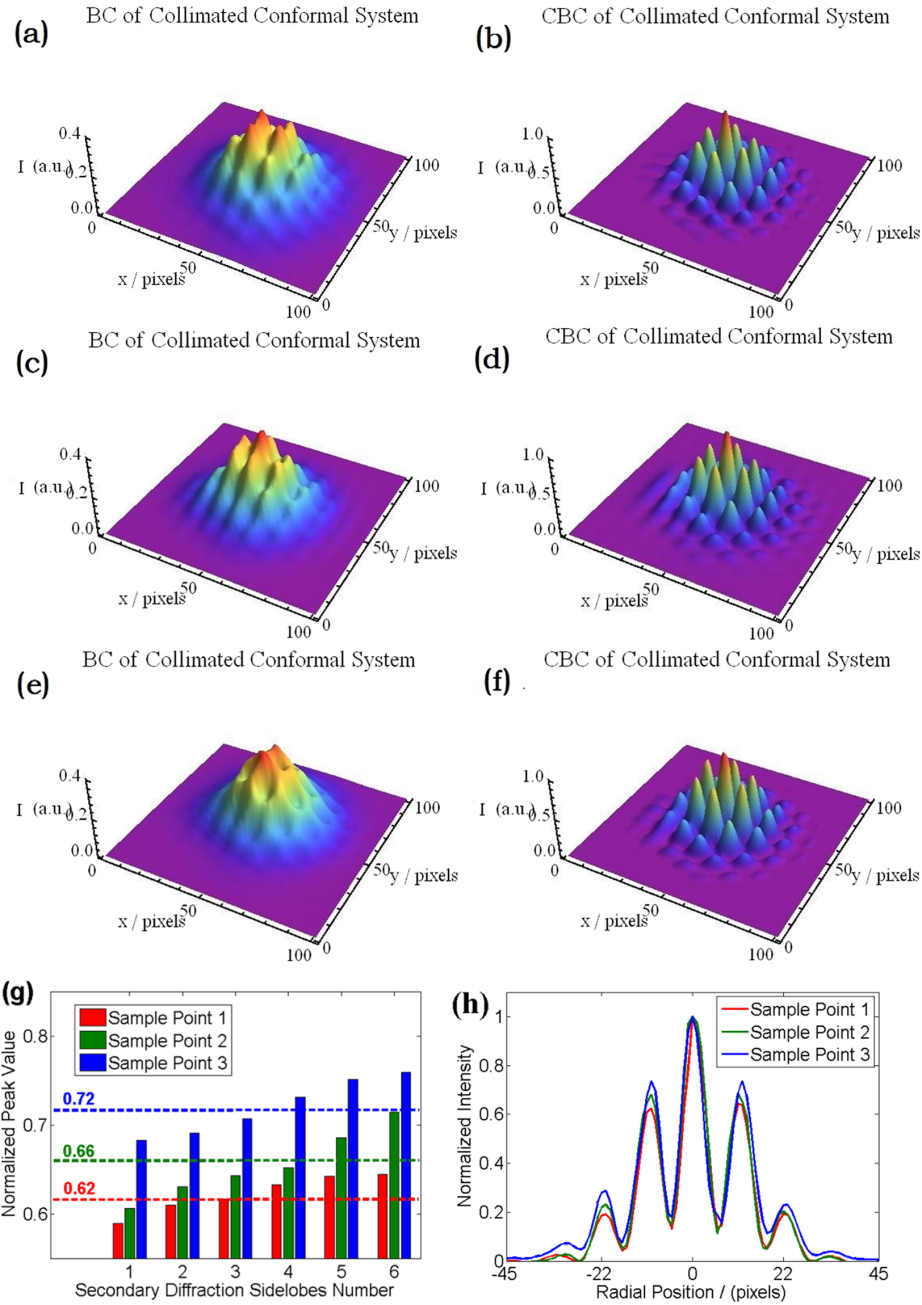
The long exposure combining beam profiles of incoherent combining and CBC based on collimated CCPS architecture. Top row: the first sampling point; Middle row: the second sampling point; Bottom row: the third sampling point.

To test the CBC performance of collimated CCPS (without defocusing) at the three chosen sampling points, a series of experimental investigations have been carried out. In our experiment, the iteration rate of CCD camera is 30 Hz, and in every case we have continuously measured and recorded 900 frames of the intensity distributions, which represent the changes of intensity distribution in 30 seconds. The superposition calculations of the 900 frames data represent the long exposure (30 seconds) intensity distributions, just as Fig. 3 shows. The validity of the active phase-control system and the differences between incoherent combining and coherent combining can be directly observed from the long exposure intensity distributions. The three dimensional long exposure (30 seconds) combining beam profiles on the CCD camera (incoherent combining and CBC experiments results of CCPS at the three sampling points mentioned above) are shown in Fig. 3. Through the previous theoretical analysis, when the overlap condition of the array beams deviates from the ideal condition, the most obvious change in the CBC result is the enhancement of the intensity peak ratio of the secondary sidelobes relative to that of the central lobe. Thus, the key point of data analysis should be focused on the variation of the intensity peak ratio of the six secondary sidelobes to the central lobe. Based on this consideration, the intensity distributions of CBC experiments at the three sampling points are normalized to 1, respectively. From the results, the fringes contrasts are calculated to be 0.87, 0.84 and 0.89 for the three sampling points, which represent all CBC experiments are effective and successful. By carefully calculations, the normalized peak values of the six secondary diffraction spots of CBC are shown in Fig. 3(g). The six secondary diffraction sidelobes are numbered from large peak value to small. The corresponding mean values of the three sampling points are 0.62, 0.66 and 0.72, which means the energy in the secondary diffraction spots increases along with the decreasing propagation distance. The standard deviations of the normalized peak values for the secondary diffraction sidelobes of the three sampling points are 0.022, 0.034 and 0.030, respectively. The differences among the six secondary diffraction sidelobes mainly come from the accuracy of piston phase-locking control system and a small amount of high order modes in the three laser beams introduced by the MFA. Moreover, the normalized intensity distributions as a function of radial positions are depicted in Fig. 3(h), which represents the intensity distributions along the horizontal straight line passing through the maximal intensity point in Fig. 3(b,d and f). By comparing the intensity distributions shown in Fig. 3(h), we find that the combining beam is spreading and the energy distribution is expanding outward to the high order diffraction levels.

**Figure 4:**
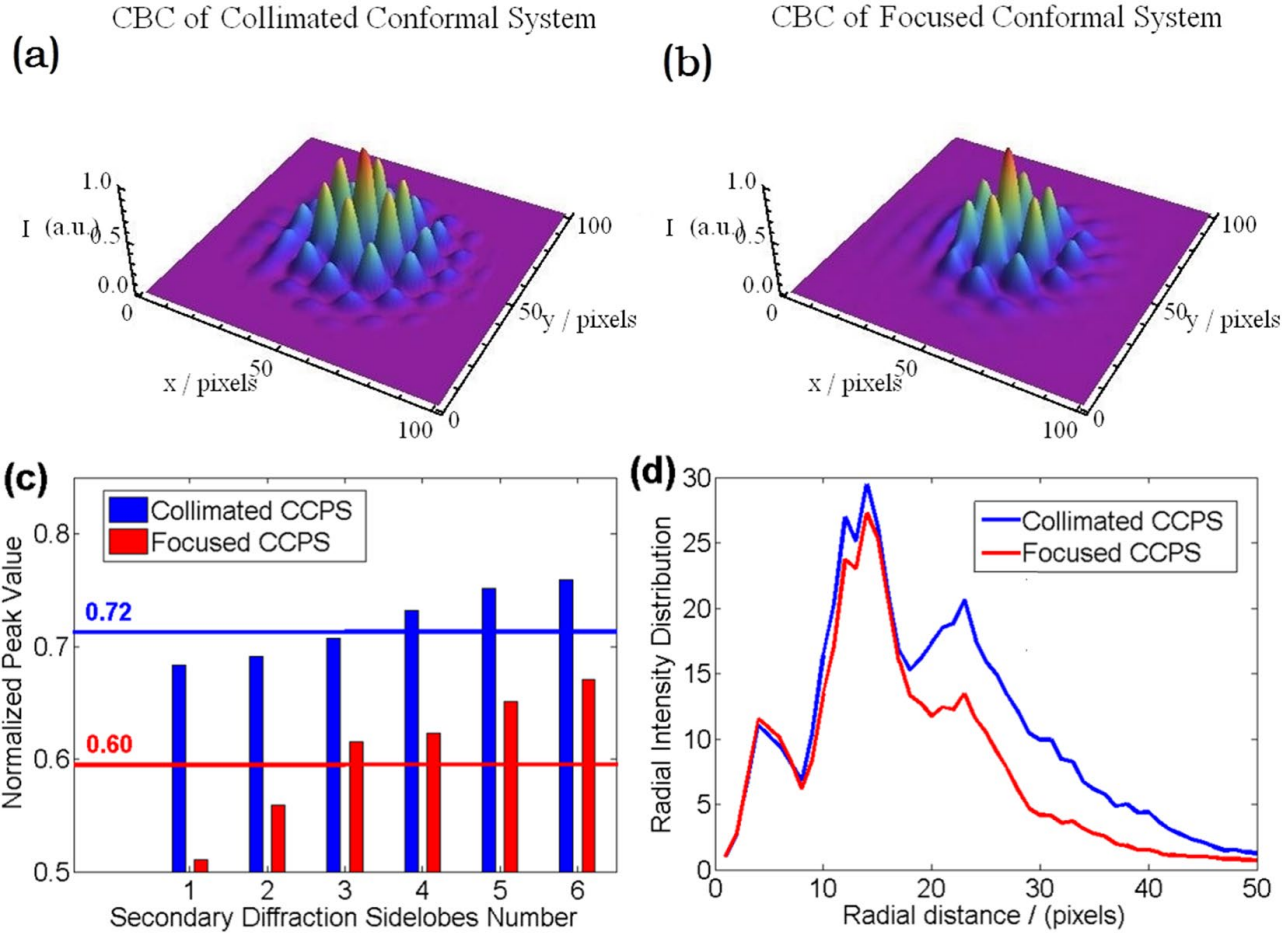
Experimental results comparisons. (**a**) and (**b**) Three dimensional intensity distributions for collimated CCPS and focused CCPS. (**c**) Normalized peak value of secondary diffraction sidelobes. (**d**) Radial intensity distributions.

Then we test the performance of the focused CCPS by applying the focusing structure at the third sampling point. Due to the limitation of the structural precision, the value of the Δ*f* is set to be 125 µm, which is an approximation of the third sampling point. The 30 seconds long exposure three dimensional beam intensity profiles of CBC experiments for the collimated CCPS and the focused CCPS are shown as Fig. 4(a,b), respectively. The fringes contrast based on focused CCPS is calculated to be 0.92. Comparing with the value of 0.89 for collimated CCPS, we can get that there is no significant differences between the fringes contrasts of the two CBC results. In spite of this, we can still get some differences from Fig. 4(a,b), such as the peak value of the secondary diffraction sidelobes and the radial energy distribution. By analyzing the experimental results, we obtain the peak values of the six secondary diffraction sidelobes, which is shown in Fig. 4(c). The ratio of the average peak intensity of the six sidelobes to the central lobe peak value is 0.72 for collimated CCPS and 0.60 for focused CCPS. Moreover, the energy distribution along the radial direction is depicted in Fig. 4(d), which shows the distribution of intensity integral values on circles with different radii. As the data of radial intensity distribution in Fig. 4(d) is employed from Fig. 4(a,b), in which the peak intensity values have been normalized to 1, the data in Fig. 4(d) is actually dimensionless and unitless. The three peak areas represent the different orders of diffraction. From the comparison shown in Fig. 4(d), we can see that the energy ratio of high orders of diffraction have been depressed significantly with the focused CCPS architecture.

**Figure 5:**
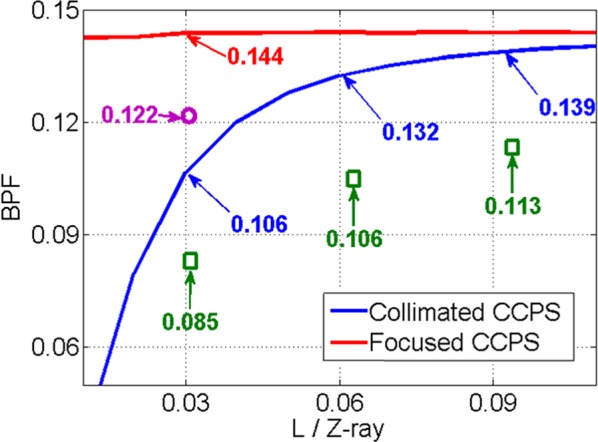
The BPF values of ideal situation and the four CBC experiments (Real lines for ideal situations by calculations, ‘’ for collimated CCPS and ‘’ for focused CCPS by experiments).

As the previous analysis, BPF can be used to evaluate the CBC effect improvement of the focused CCPS. So we define the CBC efficiency as *η* = BPF_1_/BPF_2_^7^. BPF_1_ is the power fraction of the central lobe in the experiment while BPF_2_ is the power fraction of the central lobe in the ideal situation. The theoretical calculations of the BPF_2_ values varying with the propagation distances are depicted in Fig. 5. The blue line is for collimated CCPS, while the red line is for the focused CCPS. From the experimental results based on collimated CCPS, we calculate the BPF_1_ of the three sampling points to be 0.113, 0.106 and 0.085, respectively. Comparing with the corresponding BPF_2_ values of 0.139, 0.132 and 0.106, which are pointed out by the blue arrows in Fig. 5, the CBC efficiencies at the three sampling points are 81.3%, 80.3% and 80.2%. Based on focused CCPS, the BPF_1_ of the third sampling point has improved to 0.122 with CBC efficiency of 84.7%, which is derived by dividing the corresponding BPF_2_ value of 0.144. From Fig. 5, we can see that the trend exhibited by experimental results is consistent with the theoretical curve, and is also coincident with the theoretical values in the same degrees with the calculated and aforementioned system efficiencies all above 80%.

## Discussion

We have experimentally compared the performances of collimated CCPS and focused CCPS, and demonstrated that focused CCPS can effectively improve the BPF value. Experimental results agree with the numerical calculations, achieving CBC efficiencies all above 80%. Comparing with the BPF value of collimated CCPS (0.085), the BPF value of focused CCPS improves by a factor of 44%. With its effective improvement of BPF, focused CCPS is a good scheme that can be used in high power fiber laser CBC system through a turbulent atmosphere which needs to compensate the dynamical piston and tilt phase aberrations. This work settles a fundamental requirement for CBC applications, which has great potentials in the domains of laser phased array system, target-in-the-loop technique and laser atmospheric communication systems.

## Method

### Measurement Method

A CCD camera with pixel size of 4.4 μm × 4.4 μm from Spiricon Inc. is used as a two-dimensional detector array for the patterns detection of the output combined beam. A gain adjusting PD with the rise time of 25 ns and bandwidth of 150 MHz is employed to provide the feedback signal to the algorithms controllers. Temporal domain characteristics of different situations are detected by employing a 0.5 GHz oscilloscope.
